# Tyrotoxicon

**Published:** 1917-06

**Authors:** 


### ABSTRACTS

---

**Owing to the departure from Buffalo of Major W. W. Quinton, M. D., we would be glad to have some one volunteer to abstract from several journals in the Spanish language.**

---

**Tyrotoxicon.** R. F. Ives, Brooklyn, L. I. Med. Jour., May, reports a case from ice cream, with marked cardiac degeneration and probably myocardial degeneration, indicated by faintness on slight exertion, for 3 weeks. Ultimate recovery.

